# Determination of 107 Pesticide Residues in Wolfberry with Acetate-buffered Salt Extraction and Sin-QuEChERS Nano Column Purification Coupled with Ultra Performance Liquid Chromatography Tandem Mass Spectrometry

**DOI:** 10.3390/molecules24162918

**Published:** 2019-08-12

**Authors:** Jia-Nan Chen, Yu-Jing Lian, Yi-Ran Zhou, Ming-Hui Wang, Xi-Qing Zhang, Jian-Hua Wang, Yong-Ning Wu, Ming-Lin Wang

**Affiliations:** 1 College of Food Science and Engineering, Shandong Agricultural University, Taian 271018, China; 2 Jieke Testing Service Co., Ltd., Yantai 265231, China; 3 Agricultural College, Shandong Agricultural University, Taian 271018, China; 4 China National Center for Food Safety Risk Assessment, Beijing 100017, China

**Keywords:** multi-residue, pesticide, wolfberry, Sin-QuEChERS Nano, UPLC/MS/MS

## Abstract

A multi-residue method for the determination of 107 pesticide residues in wolfberry has been developed and validated. Similar pretreatment approaches were compared, and the linearity, matrix effect, analysis limits, precision, stability and accuracy were validated, which verifies the satisfactory performance of this new method. The LODs and LOQs were in the range of 0.14–1.91 µg/kg and 0.46–6.37 µg/kg, respectively. The recovery of analytes at three fortification levels (10 µg/kg, 50 µg/kg, 100 µg/kg) ranged from 63.3–123.0%, 72.0–118.6% and 67.0–118.3%, respectively, with relative standard deviations (RSDs) below 15.0%. The proposed method was applied to the analysis of fifty wolfberry samples collected from supermarkets, pharmacies and farmers’ markets in different cities of Shandong Province. One hundred percent of the samples analyzed included at least one pesticide, and a total of 26 pesticide residues was detected in fifty samples, which mainly were insecticides and bactericide. Several pesticides with higher detection rates were 96% for acetamiprid, 82% for imidacloprid, 54% for thiophanate-methyl, 50% for blasticidin-S, 42% for carbendazim, 42% for tebuconazole and 36% for difenoconazole in wolfberry samples. This study proved the adaptability of the developed method to the detection of multiple pesticide residues in wolfberry and provided basis for the research on the risks to wolfberry health.

## 1. Introduction

*Lycium barbarum*, belonging to the Solanaceous family, also called Gouqizi in Chinese or wolfberry in English, has been widely cultivated in the northwest of China and the Mediterranean region [1,2]. Wolfberry is a kind of traditional Chinese medicine (TCM) and food homologous plant with health-protection functions. It has been widely used for the prevention and treatment of various diseases for more than thousands of years [3,4]. In recent years, wolfberry, a functional dietary supplement with nutritive value, has become increasingly prevalent in the Western countries and regions [5]. Wolfberry is rich in minerals, proteins, polysaccharides, amino acids, carotenoids, flavonoids and so on [6]. This characteristic makes it vulnerable to diseases and insect pests during its growth. Rust mite, gall mite, psylla, mealybug, aphid, anthracnose and powdery mildew are often encountered at the same time or alternately. As chemical agents for diseases and insect pests control as well as plant growth regulation, pesticides play an important role in the growth process and standardized management of wolfberry. Traditional Chinese medicine, as a gem of the Chinese nation, has a unique concept of treating diseases, which has been by degrees accepted both at home and abroad [7]. With the rapid growth of import and export trade of Chinese herbal medicines, China has developed into the largest supplier of wolfberry products [8,9,10]. Artificial planting has become an inevitable trend due to the large demand and the lack of wild wolfberry resources. Consequently, the issue of pesticide residues which are inevitably caused by the extensive use of pesticides in plant cultivation [11], has attracted wide attention of the international community [12,13,14]. Organophosphorus and pyrethroid pesticides are frequently applied to improve the quality and yield during the course of the growth of wolfberry. At present, there are more than 2000 kinds of pesticides registered in the world, of which about 500 are commonly used, while new pesticides are also being developed and applied. Biodegradation, photolysis, chemical oxidation and plant metabolism will decompose most pesticides, but there will still be a minor part of pesticide residues in plants and soil [15]. This small fraction of pesticide not only has serious adverse effects on the quality of wolfberry, but also gives rise to tremendous potential hazard to human health [16]. Simultaneously, there are increasing requirements and new challenges for pesticide residue detection technology [16,17].

Pesticide residue analysis is a complex trace analysis technology in which the pretreatment method plays a vital role in the detection process. The traditional extraction and purification technology cannot meet the requirements of modern pesticide residue analysis, especially under the condition that not only is the concentration of food pollutants getting lower [18,19], but also the matrices are becoming more and more complex [20]. Appropriate pretreatment technology can improve the sensitivity, detection range, precision and accuracy of the detection [21,22,23,24]. In recent years, mass spectrometry (MS) technology has been developing continuously, among which LC coupled with tandem MS (LC-MS/MS) is an effective and sensitive method for the detection of pesticide residues [25,26]. Common sample pretreatment techniques including liquid-liquid extraction (LLE) [27], solid phase extraction (SPE) [28,29], microwave-assisted extraction (MAE) [29,30], supercritical fluid extraction (SFE) [31], accelerated solvent extraction (ASE) [32,33,34], magnetic solid phase extraction (MSPE) [35,36], gel permeation chromatography (GPC) [37,38,39], as well as the QuEChERS method (quick, easy, cheap, effective, rugged and safe) which was firstly published by Anastassiades et al. in 2003 [40]. Nowadays, the QuEChERS protocol has been widely used for the determination of pesticide residues due to its significant advantages [41]. After the continuous improvement, two buffer versions, namely AOAC Official Method 2007.01 (acetate buffering) [42] and European Committee for Standardization (CEN) Standard Method EN 15,662 (citrate buffering) [43], were gradually formed on the basis of QuEChERS technology and widely utilized in sample pretreatment process prior to chromatographic analysis. Multi-pesticide residue analysis mostly used modified QuEChERS method [44,45,46]. Multi-walled carbon nanotubes is a new type of pretreatment adsorption material, which can effectively remove pigments and hydrophobic substances by combining with target molecules as non-covalent interaction. In recent years, it has attracted great attention in the purification process with the advantages of good chemical stability, high surface area, strong adsorption capacity, wide application range of pH value and low cost [47,48,49]. Multi-walled carbon nanotubes (MWCNTs) [50,51,52] and magnetic amino (m-MWCNTs-NH_2_) [53] or amino (MWCNTs-NH_2_) [20] modified multi-walled carbon nanotubes are extensively used as sorbent materials for dispersed solid phase extraction in the detection of multi-pesticide residues. Sin-QuEChERS Nano purification column is a new sample preparation product based on the QuEChERS method. The clean-up column with sorbents as multi-walled carbon nanotubes and PSA packed in has good purification effects of plant pigments, lipids, some sugars, sterols, phenol, wax, alkaline interferers, organic acids, etc. with the function of dehydration. With the extraction and purification combined into one-step, the loss of target components via solvent transfer can be avoided, resulting in fewer interferences during determination and longer instrument maintenance periods, meanwhile the sample preparation time is greatly saved. After the organic extract enters the liquid storage tank during the purification, the liquid level rises, and the air in the liquid storage tank is discharged through the vent hole. The Sin-QuEChERS Nano purification column is reversely guided in the downward process of the centrifuge tube, and the purified extract is stored in the liquid storage tank, then the built-in water blocking filter is bonded to the strong hydrophobic water blocking group to ensure that the aqueous solution does not contact with the purification packing and reservoir. The seal is used for sealing between the cylinder and the centrifuge tube. 

In this study, in order to monitor the quality of wolfberry in China, a method with acetate-buffered salt extraction and Sin-QuEChERS nanocolumn purification coupled with ultra performance liquid chromatography tandem mass spectrometry was developed for the determination of pesticide residues in wolfberry samples. Different versions of buffer salts for sample extraction and several SPE cartridges for clean-up were compared. The linearity, matrix effect, analysis limits, precision, stability and accuracy were validated in detail. Finally, the method was applied to determinate 50 wolfberry samples to validate the feasibility.

## 2. Results

### 2.1. Optimization and Comparison of the Extraction Procedure

**Figure 1:**
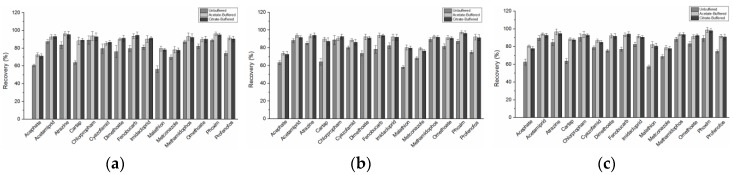
Average recoveries of 15 pesticides using the three different versions of QuEChERS (n = 5), (**a**) 10 µg/kg; (**b**) 25 µg/kg; (**c**) 50 µg/kg.

In this study, three versions of buffer salts were compared, and the extraction efficiency was evaluated using three levels (10, 25, 50 µg/kg) spiked recovery with five parallel samples. The results, were in accordance with previous studies [54], where most pesticides except for pH-sensitive ones gave excellent results when extracted with three different versions of buffer salts. As shown in Figure 1, the QuEChERS version using acetate buffering or citrate buffering more often gave higher recoveries compared with the unbuffered method for pH-dependent pesticides. For acephate, dimethoate, fenobucarb, methamidophos, omethoate and profenofos that are unstable in alkaline medium, it is easier to obtain higher recovery in buffer salt system with the pH of the matrix was maintained between 5.0 and 5.5 throughout the experiment. In addition to, as Figure 1 results demonstrate, the recovery rate of pesticides in acetate buffer version (AOAC) is slightly higher than that in citrate buffer version (CEN), but the difference is not obvious. Further, referring to previous research results [42,54], the ionization efficiency of acetonitrile and the ability of the matrix itself to interfere with acidity and alkalinity is enhanced in the acetate-buffered version, so subsequent experiments used acetate buffer version.

### 2.2. Optimization and Comparison of the Clean-up Procedure

**Figure 2:**
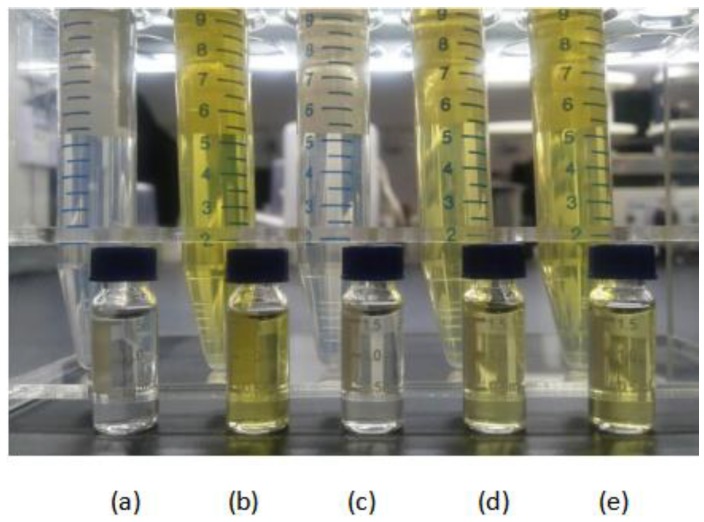
Colour comparison of extracts purified by different SPE catridges. (**a**) Sin-QuEChERS Nano; (**b**) SampliQ Florisil; (**c**) HyperSep NH_2_; (**d**) HyperSep C18; (**e**) ProElut GLASS PSA.

**Figure 3:**
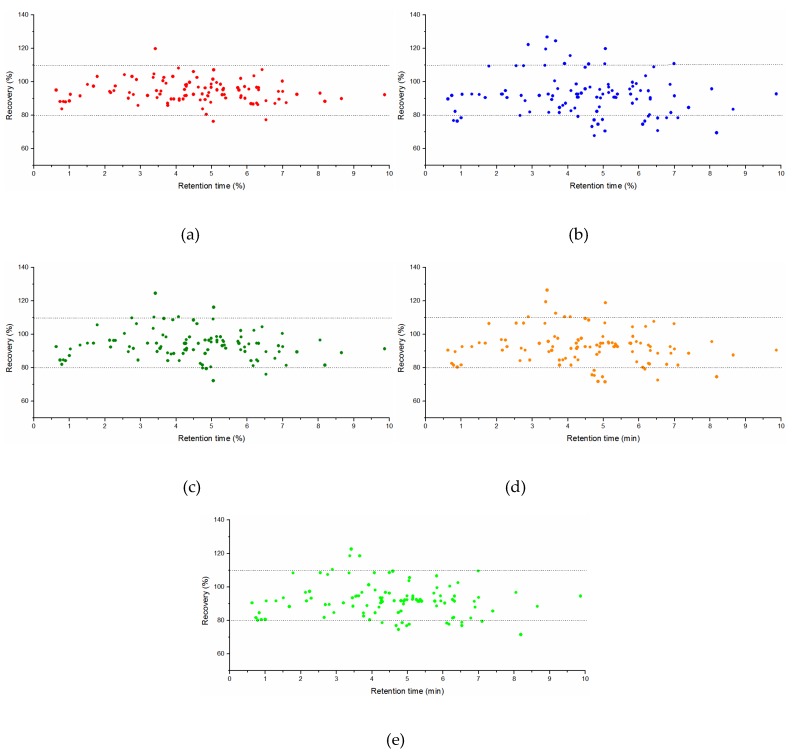
Comparison for recoveries of samples with different purification process. (**a**) Sin-QuEChERS Nano; (**b**) SampliQ Florisil; (**c**) HyperSep NH_2_; (**d**) HyperSep C18; (**e**) ProElut GLASS PSA.

Five kinds of SPE cartridges were selected for optimization and comparison of the purification procedure, and were activated by 4 mL methanol before use. Extraction solution obtained as described in Section 3.3 was passed through the SPE cartridges and 4 mL of eluent was collected for the next process which involved treatment the same as the filtrate in Section 3.3. As the Figure 2 results demonstrate vividly, compared with the other three SPE cartridges, the solution purified by the Sin-QuEChERS Nano and HyperSep NH_2_ catridges showed a colorless and transparent state. PSA was used for adsorbing pigments, organic acids, some carbohydrates and fatty acids while C_18_ and MWCNTs absorbed the pigments. The Sin-QuEChERS Nano cartridge is packed with a certain amount of MWCNTs in addition to PSA, which can better remove carotene, lutein and other colored interferents in wolfberry with less co-extracted compounds. Recoveries with the different purification processes are displayed in Figure 3, where most pesticides displayed higher recoveries for the Sin-QuEChERS Nano cartridge in comparison with other SPE columns, while all pesticides also gave satisfactory recoveries when purified by HyperSep NH_2_ columns. 

Some pesticides gave unsatisfactory recoveries (>120% or <70%) with the other three SPE cartridges owing to matrix enhancement or inhibition effects caused by matrix co-extracted compounds. Therefore, considering the recoveries, the purification effect and the simplicity of operation, Sin-QuEChERS Nano cartridges were considered the optimal choice for purification of wolfberry.

### 2.3. Method Validation

#### 2.3.1. Linearity, LOD and LOQ

The linearity was assessed by calibration curves at eight concentration levels, prepared by diluting the mixed stock solution. As shown in Table 1, the linear range of most pesticides was 5–1000 µg/kg with eight points, except for acetochlor, bendiocarb, clothianidin, cyproconazole, flutriafol, metalaxyl, propham were 2–1000 µg/kg, butralin, diethofencarb, fenbuconazole, fosthiazate, isocarbophos, phorate-sulfoxide, propiconazole were 2–500 µg/kg, and cartap, iprodione, phenthoate, phorate, thifluzamide, triflumizole were 10–1000 µg/kg. The results indicated that all targets obtained satisfactory linearity with the correlation coefficient (R^2^) of the regression curve was better than 0.9900. 

Under the optimal experimental conditions, the lowest concentration or the lowest content of the target component can be detected is considered as the detection limit (LOD). The limit of quantitation (LOQ) is the lowest concentration or the lowest quantity of the components to be measured in the sample by analytical method. The LOD and LOQ for each analytes were determined at the lowest concentration at a signal-to-noise ratio (S/N) of 3 and 10, respectively. The results were shown in Table 1 that the LOD and LOQ were 0.14–1.91 µg/kg and 0.46–6.37 µg/kg, respectively.

#### 2.3.2. Precision and Stability

In this study, the stability and precision of the established method were tested. The precision was evaluated by measuring the intra-day and inter-day variations in the relative standard deviation (RSD%) of the peak area of each analyte. In order to estimate intra-day precision, the blank spiked samples at concentration levels of 10 and 100 µg/kg were pretreated and analyzed in one day, with each spike level with five parallel samples. Inter-day precision was achieved by measuring the spiked samples at concentration levels of 10 and 100 µg/kg once a day within five consecutive days. As shown in Table 2, the relative standard deviation values (RSD) of intra-day were 0.6–9.4% (10 µg/kg) and 0.8–9.1% (100 µg/kg), inter-day were 0.8–12.6% (10 µg/kg) and 1.5–11.7% (100 µg/kg). The stability was evaluated by injecting the same volume of wolfberry spiked samples (100 µg/kg) into the UPLC/MS/MS system at 0, 2, 4, 8, 12, 18 and 24 h under the same condition in one day. The RSD values were less than 11.7 for all analytes (Table 2), which indicating that the sample solutions were stable and unchanged.

#### 2.3.3. Accuracy

The accuracy of this method was tested by the blank spike recovery experiment of 107 pesticides. And the spiking levels were 10, 50, and 100 µg/kg, respectively, meanwhile the determination had five parallel samples for each spiking level. Average recoveries of 107 kinds of pesticides at three fortification levels using the analytical procedure, as presented in Table 2, were 63.3–123.0%, 72.0–118.6%, and 67.0–118.3%, respectively, which were within the range of the acceptable values. Under the premise of ensuring the accuracy and reproducibility of the results, a better recovery rate can be obtained, which is fully in line with the AOAC 2007.01 and EN 15,662 standards. All samples were analyzed on the same day and the RSD values below 15.0% at the three fortification levels indicated the accuracy was acceptable.

### 2.4. Matrix Effects

Matrix effects are caused by the co-elution of matrix constituents that play an important role in multi-residue analysis of pesticides, which can affect the ionization efficiency of target pesticides [55,56] and then influence the quantitative results by matrix enhancement and attenuation effects caused by quality of chromatographic separation, the ionization type, the amount and the type of the sample matrix, and sample preparation procedure [57,58]. For complex matrix like wolfberry is wealthy in mineral substance, proteins, polysaccharose, amino acids, carotinoid, flavonoids and so on [6], it is critical to eliminate or attenuate matrix effects by minimizing matrix co-extractives through the sample preparation procedure. In this research, the matrix effect (ME) was calculated by the following equation:

(1) $$\mathrm{ME}\;=\frac{A_{\mathrm{Matrix}}}{A_{s}}\;\times \;100\%$$

where A_Matrix_ is the peak area of matrix standard sample and A_s_ is the peak area of pure solvent standard sample. The matrix effect of the individual pesticide was studied at the level of 10 µg/kg with five parallel samples, which was deemed to be ignored if the ME value is between 90% and 110%, while it was regarded to be matrix suppression or enhancement effect when the value was less than 90% or greater than 110%, respectively [55,58,59]. The results showed that 71.96% of the pesticides presented a negligible ME, whereas 27.10% of the analytes presented matrix suppression effect, and only one pesticide (carbofuran) showed a matrix enhancement effect. Table 2 shows the specific ME values of each pesticide. Most of the compounds showed negligible matrix effect or mild matrix inhibition effect, suggesting that this method was suitable for the determination of 107 pesticide residues in wolfberry. However, there are still several pesticides with strong matrix effect: ME < 80% (boscalid, hexythiazox, metconazole) or ME > 120% (carbofuran). Therefore, to overcome and compensate for these matrix effects, matrix-matched standard curves were used in the quantitative analysis.

### 2.5. Real Samples Analysis

**Figure 4:**
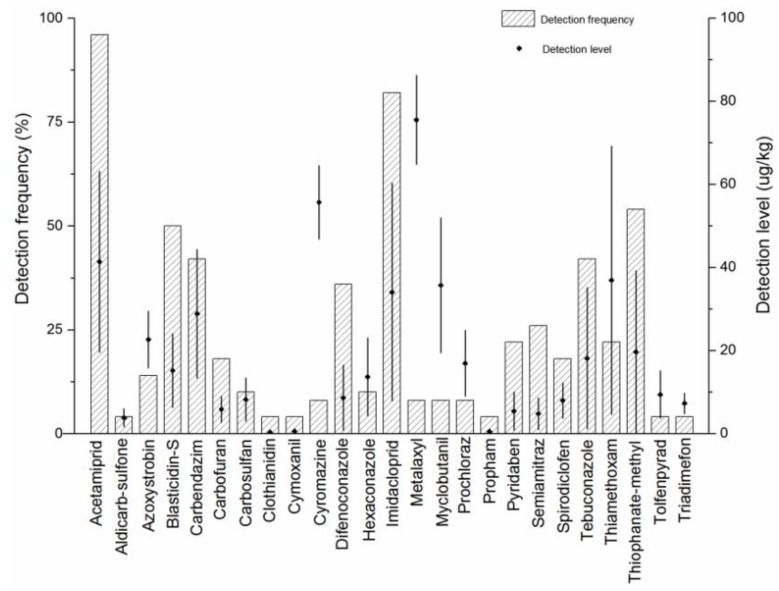
Detection results of pesticide residues in real samples with the developed method.

The applicability of the developed method was evaluated by analyzing a total of fifty wolfberry samples collected from supermarkets, pharmacies and farmers’ markets in different cities of Shandong Province and three replicates of each sample were analysed. One hundred percent of the samples analyzed included at least one pesticide, and a total of 26 pesticide residues was detected in fifty samples, which mainly were insecticides and bactericides. Ninety-six percent of the samples was found to be acetamiprid with concentration ranging from 19.54 to 63.15 µg/kg, and imidacloprid was detected in eighty-two percent of the samples with concentration ranging from 7.80 to 60.26 µg/kg. In addition, several pesticides with higher detection frequency in wolfberry were thiophanate-methyl (54%), blasticidin-S (50%), carbendazim (42%), tebuconazole (42%) and difenoconazole (36%). The detection frequency of cyromazine and metalaxyl in wolfberry is only 8%, but the detection concentration is as high as 46.78–64.56 µg/kg and 64.75–86.22 µg/kg, respectively. Moreover, carbendazim (44.36 µg/kg), myclobutanil (51.95 µg/kg) and thiamethoxam (69.21 µg/kg) were found higher concentration in some samples occasionally. All pesticide residues detected in wolfberry were below the MRLs specified by the EU and China, except for pesticides without MRLs. The residue level of imidacloprid with a detection frequency of up to 82% was lower than the MRL (1 mg/kg) set by China (GB 2763-2016). Figure 4 reveals the types, frequency and concentration range of 26 pesticides detected in 50 wolfberry samples. From this test, the adaptability of the developed method to the detection of multiple pesticide residues in wolfberry was determined.

## 3. Materials and Methods

### 3.1. Chemicals and Reagents

Pesticide reference standards of all analytes were of purity > 98%, purchased from Aldrich-Sigma (Shanghai, China) and Dr. Ehrenstorfer GmbH (Augsburg, Germany). Each kind of pesticide standard substances was accurately labeled as 10 mg in a 100 mL volumetric flask, then dissolved with methanol and fixed to the scale line. A composite sample working standard solution was prepared by combining aliquots of each stock solution and diluting in methanol to obtain a final concentration of 10 mg/L, a series of matrix-matched standard solutions was obtained by gradually diluting the stock solution with blank matrix solution. All solutions were stored at 4 °C before use.

Acetonitrile (MeCN) and methanol (MeOH) were of HPLC grade, purchased from Fisher Scientific (Pittsburgh, PA, USA). High purity water was obtained using a Milli-Q POD water purification system (Millipore, Schwalbach, Germany). LUMTECH^TM^ Sin-QuEChERS Nano catridges (2 g Na_2_SO_4_, 0.6 g MgSO_4_, 90 mg PSA, 15 mg MWCNTs) and the three kinds of salting out packages for the traditional QuEChERS method (4 g MgSO_4_, 1 g NaCl), AOAC 2007.01 Method (6 g MgSO_4_, 1.5 g NaOAc) and EN 15,662 Method (4 g MgSO_4_, 1 g NaCl, 1 g TSCD, 0.5 g DHS) respectively were provided by Lumiere Technologies (Beijing, China). HyperSep C18 and HyperSep NH2 solid phase extraction catridges were purchased from Thermo Scientific (Waltham, MA, USA), SampliQ Florisil solid phase extraction catridges were obtained from Agilent Technologies (Santa Clara, CA, USA), and ProElut GLASS PSA solid phase extraction catridges were obtained from Dikma Technologies (Beijing, China), respectively.

### 3.2. Instrument

An ACQUITY Quattro Premier XE system (Waters Corp., Milford, MA, USA) was used for analysis. Chromatographic separation was performed at 35 °C with an ACQUITY UPLC HSS T3 (1.8 μm, 2.1 × 100 mm, Waters Corp., Milford, MA, USA). The mobile phase A was 0.1% formic acid solution and the mobile phase B was 0.1% formic acid acetonitrile. Initial composition of the mobile phase was 80% of solvent A and 20% of solvent B, reaching the 95% of solvent B at 6 min. The concentration of B remained at 95% for 2 min before returning to the initial state in 1 min. Re-equilibration of the column was performed for 1 min before the next injection was conducted. The flow rate of the eluent was 0.4 mL/min and the injection volume was 1 μL. The mass spectrometer was equipped with an electrospray ionization (ESI) interface operating at positive (ESI+) or negative (ESI−) mode, and MRM mode was adopted for date collection. The electrospray voltage, the atomizing gas pressure, the auxiliary airflow speed and the ion source temperature were set as 3000 V, 7.0 Bar, 150 L/h and 400 °C, respectively. The 107 analytical parameters of liquid chromatography tandem mass spectrometry are listed in Table 1.

### 3.3. Sample Preparation

Wolfberry samples were collected from supermarkets, pharmacies and farmers’markets in different cities of Shandong Province, then the samples were ground into powder, passed through mesh screen (0.42 mm). The prepared samples were stored at 4 ℃ and analysed withinn 24 h following the procedure described below. Wolfberry (pesticide-free) obtained from an organic production base was used as blank matrix for preparing standard curve and the recovery studies.

**Figure 5:**
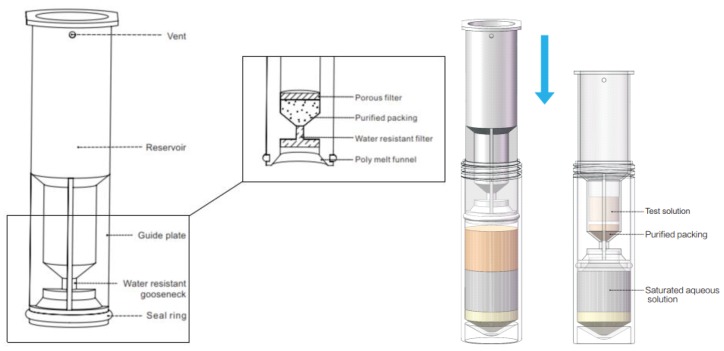
A schematic diagram of the structure and use of Sin-QuEChERS Nano catridges.

**Figure 6:**
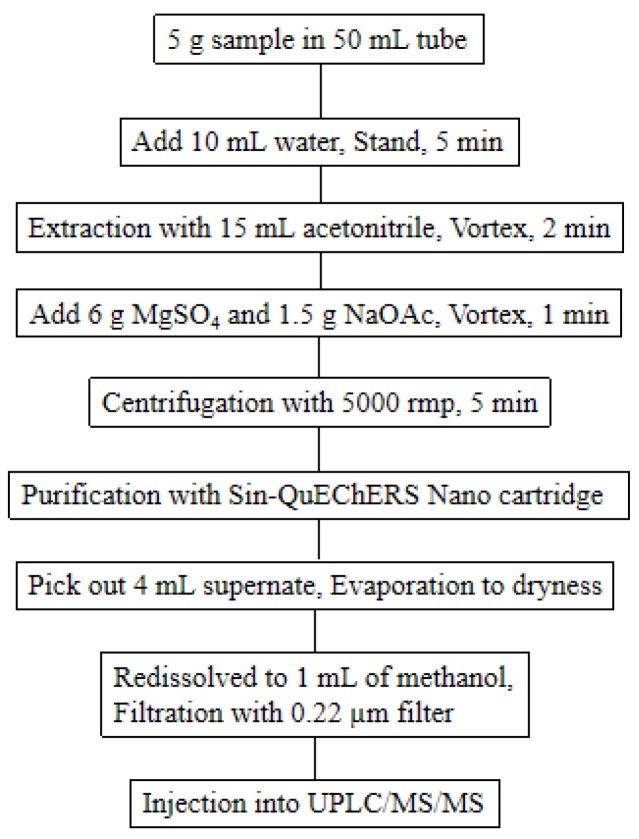
Scheme of pesticide extraction procedures from wolfberry samples.

Five grams of ground wolfberry sample were weighed exactly into a 50 mL polypropylene centrifuge tube and soaked with 10 mL water for 5 min. Subsequently, 15 mL acetonitrile was added, next vortexed for 2 min, after following AOAC 2007. 01 extraction salt pack was added, after that vortexed for 1 min, succeeding centrifuged at 5000 rpm for 5 min. The Sin-QuEChERS Nano purification tube was taken into the 50 mL centrifuge tube, which was filled with extraction liquid, eventually the purification tube was slowly pressed down by an automatic apparatus to making up about 4 mL supernatant in the storage tank of the purification pipe. The structure and usage of Sin-QuEChERS Nano catridges were shown in Figure 5. The supernatant of 4 mL was accurately transferred to 10 mL centrifugal tube, dried with nitrogen gas stream and resolved with 1 mL methanol. Before UPLC/MS/MS analysis, the solution was filtered through a 0.22 µm filter (Dikma, Technologies). The flow chart of sample preparation procedure is presented in Figure 6.

## 4. Conclusions

In this work, a simple and rapid method has been developed for the determination of 107 pesticide residues in wolfberry samples using UPLC/MS/MS analysis. The qualitative and quantitative analysis of 107 pesticides can be accomplished in 10 min at a time with one injection. The Sin-QuEChERS Nano purification column can not only effectively remove pigments, organic acids, alkaline interferents, fat and water, but also save sample preparation time and avoid the losses caused by solvent transfers. It greatly simplifies the purification process of the samples, while improving the detection efficiency and accuracy. The linearity, matrix effect, analysis limits, precision, stability and accuracy were validated in detail. The LODs and LOQs of the target pesticides obtained by this method were in the range of 0.14–1.91 µg/kg and 0.46–6.37 µg/kg, respectively. Under the premise of ensuring the accuracy and reproducibility of the results, the average recoveries (80%–120%) and RSD (<15%) of most target pesticides at the three spike levels were acceptable, which met the requirements of conventional pesticide residues screening. Fifty commercial wolfberry samples were tested for pesticide residues by the developed method, all of which were positive, and 26 pesticides were detected. These results demonstrated that the proposed method was sensitive, fast, simple and reliable for the simultaneous determination of 107 pesticide residues in wolfberry, furthermore did not require complex purification process to allow routine analysis of a large quantity of samples. Furthermore, the developed approach can further expand the types of target pesticides and be applied to the detection of pesticide residues in more other traditional Chinese medicine. This will be the focus of our future work.

## Figures and Tables

**Table 1:** Liquid chromatography tandem mass spectrometry analysis parameters, LODs, LOQs, Linear range and Calibration Curve Coefficients (R2) of 107 pesticides.

No.	Compound	Elemental composition	Precursor ion	Retention Time (min)	Precursor ions (*m/z*)	Products (*m/z*)	Cone voltage (V)	Collision energy (qv)	LOD (µg/kg)	LOQ (µg/kg)	Linear range (µg/kg)	R^2^
1	Abamectin	C_48_H_72_O_14_	[M + NH_4_]^+^	8.65	890.7	305.1	18	22	1.48	3.94	5–1000	0.9998
					890.7	567.2	18	10				
2	Acephate	C_4_H_10_NO_3_PS	[M + H]^+^	0.79	184.0	49.2	10	16	1.17	3.91	5–1000	0.9981
3	Acetamiprid	C_10_H_11_ClN_4_	[M + H]^+^	2.29	223.0	56.1	22	10	0.46	1.54	5–1000	0.9999
					223.0	126.0	22	12				
4	Acetochlor	C_14_H_20_ClNO_2_	[M + H]^+^	5.14	270.0	148.1	15	20	0.27	0.90	2–1000	0.9995
					270.0	224.0	15	10				
5	Aldicarb	C_7_H_14_N_2_O_2_S	[M + Na]^+^	2.80	213.1	89.1	25	16	0.45	1.50	5–1000	0.9697
					213.1	116.1	25	11				
6	Aldicarb-Sulfone	C_7_H_14_N_2_O_4_S	[M + H]^+^	1.30	223.0	86.0	25	10	0.72	2.39	5–1000	0.9990
					223.0	148.0	25	8				
7	Aldicarb-Sulfoxide	C_7_H_14_N_2_O_3_S	[M + H]^+^	0.89	207.1	89.0	10	14	0.68	2.27	5–1000	0.9911
					207.1	132.0	10	5				
8	Ametryn	C_9_H_17_N_5_S	[M + H]^+^	2.89	228.1	68.1	25	25	0.50	1.66	5–1000	0.9998
					228.1	186.0	25	10				
9	Atrazine	C_8_H_14_ClN_5_	[M + H]^+^	3.66	216.1	96.1	22	18	0.90	3.00	5–1000	0.9996
					216.1	174.1	22	14				
10	Azoxystrobin	C_22_H_17_N_3_O_5_	[M + H]^+^	4.63	404.0	329.0	25	25	0.33	1.08	5–1000	0.9995
					404.0	372.0	25	12				
11	Bendiocarb	C_11_H_13_NO_4_	[M + H]^+^	3.38	224.1	109.0	17	18	0.24	0.79	2–1000	0.9987
					224.1	167.0	17	8				
12	Blasticidin-S	C_17_H_26_N_8_O_5_	[M + H]^+^	2.76	423.5	189.3	18	12	0.98	3.27	5–1000	0.9994
					423.5	210.6	18	12				
13	Boscalid	C_18_H_12_Cl_2_N_2_O	[M − H]^−^	4.75	342.9	139.9	32	20	0.60	2.01	5–1000	0.9994
					342.9	307.0	32	20				
14	Bupirimate	C_13_H_24_N_4_O_3_S	[M + H]^+^	5.06	317.0	108.0	31	28	0.48	1.61	5–1000	0.9995
					317.0	166.0	31	28				
15	Buprofezin	C_16_H_23_N_3_OS	[M + H]^+^	5.36	306.1	116.0	20	10	0.82	2.74	5–1000	0.9997
					306.1	201.0	20	10				
16	Butralin	C_14_H_21_N_3_O_4_	[M + H]^+^	8.19	296.3	222.2	20	20	0.21	0.70	2–500	0.9997
					296.3	240.3	20	17				
17	Carbaryl	C_12_H_11_NO_2_	[M + H]^+^	3.58	202.0	117.0	19	28	0.68	2.26	5–1000	0.9998
					202.0	145.0	19	22				
18	Carbendazim	C_9_H_9_N_3_O_2_	[M + H]^+^	6.32	192.0	132.0	17	22	1.11	3.70	5–1000	0.9996
					192.0	160.0	17	22				
19	Carbofuran	C_12_H_15_NO_3_	[M + H]^+^	3.42	222.1	123.0	22	12	0.71	2.38	5–1000	0.9991
					222.1	165.1	22	12				
20	Carbosulfan	C_20_H_32_N_2_O_3_S	[M + H]^+^	4.27	381.1	117.9	24	18	0.96	3.20	5–1000	0.9987
					381.1	159.9	24	18				
21	Cartap	C_7_H_15_N_3_O_2_S_2_	[M + H]^+^	0.63	238.0	61.1	25	22	1.57	5.22	10–1000	0.9999
					238.0	105.0	25	16				
22	Chlorantraniliprole	C_18_H_14_BRCl_2_N_5_O_2_	[M + H]^+^	4.20	484.1	286.1	25	20	0.42	1.39	5–1000	0.9998
					484.1	453.0	25	18				
23	Chlorfenvinphos	C_12_H_14_Cl_3_O_4_P	[M − H]^−^	5.30	358.9	99.0	20	30	0.96	3.22	5–1000	0.9989
					358.9	155.0	20	12				
24	Chlorpropham	C_10_H_12_ClNO_2_	[M + H]^+^	4.85	214.6	126.0	20	15	0.72	2.41	5–1000	0.9983
					214.6	154.0	20	10				
25	Clodinafop-propargyl	C_17_H_13_ClFNO_4_	[M + H]^+^	6.78	350.0	266.0	22	20	0.86	2.87	5–1000	0.9997
					350.0	91.0	22	18				
26	Clothianidin	C_6_H_8_ClN_5_O_2_S	[M + H]^+^	2.16	249.9	132.0	22	15	0.22	0.74	2–1000	0.9995
					249.9	169.0	22	10				
27	Cyantraniliprole	C_19_H_14_BrClN_6_O_2_	[M + H]^+^	3.77	475.0	286.0	25	15	0.31	1.03	5–1000	0.9995
					475.0	444.0	25	15				
28	Cyazofamid	C_13_H_13_ClN_4_O_2_S	[M + H]^+^	5.34	325.0	107.9	20	20	0.36	1.19	5–1000	0.9991
					325.0	261.0	20	10				
29	Cymoxanil	C_7_H_10_N_4_O_3_	[M + H]^+^	2.55	199.0	111.0	15	18	0.86	2.87	5–1000	0.9989
					199.0	128.0	15	8				
30	Cyproconazole	C_15_H_18_ClN_3_O	[M + H]^+^	5.32	292.2	70.2	30	18	0.22	0.73	2–1000	0.9996
					292.2	125.1	30	24				
31	Cyprodinil	C_14_H_15_N_3_	[M + H]^+^	4.09	226.0	93.0	30	26	0.84	2.81	5–1000	0.9992
					226.0	108.0	30	20				
32	Cyromazine	C_6_H_10_N_6_	[M + H]^+^	2.66	167.2	60.0	28	20	1.03	3.43	5–1000	0.9992
					167.2	108.0	28	20				
33	Diazinon	C_12_H_21_N_2_O_3_PS	[M + H]^+^	6.99	305.1	96.9	30	35	0.63	2.10	5–1000	0.9990
					305.1	169.0	30	22				
34	Diethofencarb	C_14_H_21_NO_4_	[M + H]^+^	4.49	268.3	124.0	19	40	0.26	0.86	2–500	0.9989
					268.3	226.0	19	10				
35	Difenoconazole	C_19_H_17_Cl_2_N_3_O_3_	[M + H]^+^	5.40	406.0	111.1	20	60	0.60	1.99	5–1000	0.9994
					406.0	251.1	20	25				
36	Dimethoate	C_5_H_12_NO_3_PS_2_	[M + H]^+^	2.25	230.1	125.0	22	10	0.40	1.34	5–1000	0.9991
					230.1	199.0	22	6				
37	Dimethomorph	C_21_H_22_ClNO_4_	[M + H]^+^	4.25	388.1	165.0	35	30	0.20	0.65	5–1000	0.9994
					388.1	300.9	35	20				
38	Diniconazole	C_15_H_17_Cl_2_N_3_O	[M + H]^+^	6.30	326.1	70.2	37	25	0.71	2.38	5–1000	0.9994
					326.1	159.0	37	34				
39	Dinotefuran	C_7_H_14_N_4_O_3_	[M + H]^+^	1.00	203.0	129.0	13	12	0.86	2.88	5–1000	0.9983
					203.0	156.9	13	6				
40	Diuron	C_9_H_10_Cl_2_N_2_O	[M + H]^+^	3.76	233.0	46.3	28	14	0.27	0.91	5–1000	0.9974
					233.0	72.1	28	18				
41	Epoxiconazole	C_17_H_13_ClFN_3_O	[M + H]^+^	4.68	330.0	101.0	25	50	0.55	1.84	5–1000	0.9997
					330.0	121.0	25	22				
42	Ethion	C_9_H_22_O_4_P_2_S_4_	[M + H]^+^	8.05	384.9	97.0	30	46	0.80	2.66	5–1000	0.9994
					384.9	199.1	30	10				
43	Fenbuconazole	C_19_H_17_ClN_4_	[M + H]^+^	4.89	337.0	70.1	32	20	0.24	0.82	2–500	0.9990
					337.0	125.0	32	36				
44	Fenhexamid	C_14_H_17_Cl_2_NO_2_	[M + H]^+^	4.74	302.1	55.3	32	38	0.34	1.15	5–1000	0.9998
					302.1	97.2	32	22				
45	Fenobucarb	C_12_H_17_NO_2_	[M + H]^+^	4.38	208.0	94.9	16	14	0.87	2.89	5–1000	0.9966
					208.0	152.0	16	8				
46	Flonicamid	C_9_H_6_F_3_N_3_O	[M + H]^+^	1.78	230.2	148.1	35	25	1.36	4.52	5–1000	0.9992
					230.2	203.1	35	15				
47	Fluazifop-butyl	C_19_H_20_F_3_NO_4_	[M + H]^+^	6.32	384.1	282.1	28	20	0.45	1.49	5–1000	0.9985
					384.1	328.1	28	14				
48	Fluazinam	C_13_H_4_Cl_2_F_6_N_4_O_4_	[M + H]^+^	6.17	465.0	338.1	23	47	0.27	0.91	5–1000	0.9997
					465.0	373.0	23	26				
49	Fluopicolide	C_14_H_8_Cl_3_F_3_N_2_O	[M + H]^+^	4.82	386.2	173.0	35	25	0.43	1.45	5–1000	0.9995
					386.2	175.0	35	25				
50	Flusilazole	C_16_H_15_F_2_N_3_Si	[M + H]^+^	5.93	316.0	165.0	30	28	0.42	1.39	5–1000	0.9993
					316.0	247.0	30	18				
51	Flutriafol	C_16_H_13_F_2_N_3_O	[M + H]^+^	4.24	302.1	70.2	30	18	0.14	0.46	2–1000	0.9999
					302.1	123.1	30	29				
52	Fosthiazate	C_9_H_18_NO_3_PS_2_	[M + H]^+^	3.63	284.0	104.0	20	18	0.20	0.68	2–500	0.9998
					284.0	228.0	20	6				
53	Haloxyfop-methyl	C_16_H_13_ClF_3_NO_4_	[M + H]^+^	5.82	376.0	91.1	25	25	0.23	0.77	5–1000	0.9998
					376.0	316.1	25	10				
54	Hexaconazole	C_14_H_17_Cl_2_N_3_O	[M + H]^+^	6.05	315.0	70.1	31	22	0.46	1.52	5–1000	0.9992
					315.0	159.0	31	28				
55	Hexythiazox	C17H21ClN2O2S	[M + H]^+^	6.53	353.0	168.1	24	26	0.90	2.98	5–1000	0.9995
					353.0	228.1	24	14				
56	Imazalil	C_14_H_14_Cl_2_N_2_O	[M + H]^+^	2.93	296.9	158.9	25	20	0.60	2.00	5–1000	0.9999
					296.9	201.0	25	15				
57	Imidacloprid	C_9_H_10_ClN_5_O_2_	[M + H]^+^	2.13	256.1	175.1	22	20	0.31	1.02	5–1000	0.9999
					256.1	209.1	22	15				
58	Indoxacarb	C_22_H_17_ClF_3_N_3_O_7_	[M + H]^+^	5.83	528.0	150.0	25	22	0.37	1.23	5–1000	0.9993
					528.0	203.0	25	40				
59	Iprodione	C_13_H_13_Cl_2_N_3_O_3_	[M + H]^+^	4.98	330.0	244.7	15	16	1.57	5.23	10–1000	0.9995
					330.0	288.0	15	15				
60	Isocarbophos	C_11_H_16_NO_4_PS	[M + H]^+^	4.30	291.1	121.1	12	30	0.21	0.70	2–500	0.9992
					291.1	231.1	12	13				
61	Isoprocarb	C_11_H_15_NO_2_	[M + H]^+^	3.91	194.1	95.1	18	14	0.33	1.09	5–1000	0.9981
					194.1	137.1	18	8				
62	Isoprothiolane	C_12_H_18_O_4_S_2_	[M + H]^+^	5.04	291.1	188.8	15	18	0.22	0.72	5–1000	0.9991
					291.1	230.9	15	10				
63	Isoproturon	C_12_H_18_N_2_O	[M + H]^+^	3.72	207.0	72.0	28	22	0.30	0.99	5–1000	0.9982
					207.0	165.1	28	15				
64	Malathion	C_10_H_19_O_6_PS_2_	[M + H]^+^	4.99	331.0	99.0	14	24	0.21	0.71	5–1000	0.9998
					331.0	127.0	14	12				
65	Metalaxyl	C_15_H_21_NO_4_	[M + H]^+^	4.49	280.1	192.1	30	17	0.21	0.70	2–1000	0.9997
					280.1	220.1	30	13				
66	Metconazole	C_17_H_22_ClN_3_O	[M + H]^+^	5.05	320.1	70.0	25	22	0.52	1.73	5–1000	0.9993
					320.1	125.0	25	36				
67	Methamidophos	C_2_H_8_NO_2_PS	[M + H]^+^	0.74	142.0	94.0	22	10	0.44	1.48	5–1000	0.9999
					142.0	124.9	22	10				
68	Methomyl	C_5_H_10_N_2_O_2_S	[M + H]^+^	1.51	163.0	88.0	20	10	0.32	1.06	5–1000	0.9992
					163.0	106.0	20	10				
69	Methoxyfenozide	C_22_H_28_N_2_O_3_	[M + H]^+^	4.90	369.1	149.1	25	18	0.53	1.78	5–1000	0.9994
					369.1	313.2	25	8				
70	Metolachlor	C_15_H_22_ClNO_2_	[M + H]^+^	6.27	284.1	176.1	30	25	0.40	1.35	5–1000	0.9997
					284.1	252.1	30	15				
71	Metribuzin	C_8_H_14_N_4_OS	[M + H]^+^	3.86	215.0	131.0	10	18	0.62	2.07	5–1000	0.9987
					215.0	89.0	20	16				
72	Myclobutanil	C_15_H_17_ClN_4_	[M + H]^+^	4.59	289.1	70.2	28	18	0.35	1.18	5–1000	0.9992
					289.1	125.1	28	32				
73	Omethoate	C_5_H_12_NO_4_PS	[M + H]^+^	0.83	214.0	125.0	20	22	0.63	2.09	5–1000	0.9983
					214.0	183.0	20	11				
74	Paclobutrazol	C_15_H_20_ClN_3_O	[M + H]^+^	5.15	294.1	70.2	30	20	0.40	1.34	5–1000	0.9995
					294.1	125.1	30	38				
75	Penconazole	C_13_H_15_Cl_2_N_3_	[M + H]^+^	4.99	284.0	70.1	28	16	0.38	1.26	5–1000	0.9999
					284.0	159.0	28	34				
76	Phenthoate	C_12_H_17_O_4_PS_2_	[M + H]^+^	6.88	321.0	135.0	30	20	1.91	6.37	10–1000	0.9999
					321.0	163.0	30	12				
77	Phorate	C_7_H_17_O_2_PS_3_	[M + H]^+^	5.82	261.0	75.0	10	12	1.62	5.40	10–1000	0.9949
78	Phorate-sulfone	C_7_H_17_O_4_PS_3_	[M + NH_4_]^+^	4.29	293.0	115.0	16	24	0.51	1.71	5–1000	0.9979
					293.0	171.0	16	6				
79	Phorate-sulfoxide	C_7_H_17_O_3_PS_3_	[M + H]^+^	3.55	277.0	96.9	18	32	0.15	0.50	2–500	0.9989
					277.0	143.0	18	20				
80	Phoxim	C_12_H_15_N_2_O_3_PS	[M + H]^+^	5.77	299.0	129.0	12	13	0.45	1.49	5–1000	0.9977
					299.0	153.0	12	7				
81	Piperonyl-butoxide	C_19_H_30_O_5_	[M + NH_4_]^+^	6.27	356.3	119.0	20	30	0.57	1.91	5–1000	0.9999
					356.3	176.9	20	10				
82	Pirimiphos-methyl	C_11_H_20_N_3_O_3_PS	[M + H]^+^	6.90	306.1	108.1	30	32	0.55	1.84	5–1000	0.9991
					306.1	164.1	30	22				
83	Prochloraz	C_15_H_16_Cl_3_N_3_O_2_	[M + H]^+^	9.87	376.1	266.0	22	10	0.89	2.97	5–1000	0.9978
					376.1	308.0	22	10				
84	Profenofos	C_11_H_15_BrClO_3_PS	[M − H]^−^	7.40	372.9	127.9	30	40	1.14	3.80	5–1000	0.9999
					372.9	302.6	30	20				
85	Prometryn	C_10_H_19_N_5_S	[M + H]^+^	3.45	242.0	158.0	20	16	0.69	2.31	5–1000	0.9995
					242.0	200.1	20	12				
86	Propham	C_10_H_13_NO_2_	[M + H]^+^	4.07	180.0	77.0	18	20	0.26	0.88	2–1000	0.9991
					180.0	120.0	18	10				
87	Propiconazole	C_15_H_17_Cl_2_N_3_O_2_	[M + H]^+^	5.15	342.0	69.0	25	20	0.16	0.54	2–500	0.9996
					342.0	159.0	25	30				
88	Propoxur	C_11_H_15_NO_3_	[M + H]^+^	3.36	210.0	111.0	12	16	0.44	1.45	5–1000	0.9968
89	Pyridaben	C_19_H_25_ClN_2_OS	[M + H]^+^	7.00	365.1	147.0	22	20	0.24	0.80	5–1000	0.9985
					365.1	309.1	22	8				
90	Semiamitraz	C_10_H_14_N_2_	[M + H]^+^	3.94	163.0	96.3	18	10	1.32	4.40	5–1000	0.9921
					163.0	118.4	18	10				
91	Sethoxydim	C_17_H_29_NO_3_S	[M + H]^+^	6.42	328.3	254.3	23	15	1.50	4.99	5–1000	0.9976
					328.3	282.0	23	10				
92	Simazine	C_7_H_12_ClN_5_	[M + H]^+^	3.47	202.0	96.0	25	20	0.30	0.99	5–1000	0.9999
					202.0	124.0	25	14				
93	Spirodiclofen	C_21_H_24_Cl_2_O_4_	[M + H]^+^	7.10	411.1	71.2	25	13	0.78	2.61	5–1000	0.9997
					411.1	313.0	25	13				
94	Tebuconazole	C_16_H_22_ClN_3_O	[M + H]^+^	4.82	308.0	70.1	34	22	0.24	0.79	5–1000	0.9996
					308.0	125.0	34	40				
95	Tebufenozide	C_22_H_28_N_2_O_2_	[M + H]^+^	5.25	353.0	105.0	12	22	0.28	0.95	5–1000	0.9996
					353.0	133.0	12	18				
96	Thiabendazole	C_10_H_7_N_3_S	[M + H]^+^	1.03	202.0	131.0	25	25	0.30	0.99	5–1000	0.9998
					202.0	175.0	25	20				
97	Thiacloprid	C_10_H_9_ClN_4_S	[M + H]^+^	2.69	253.0	90.1	25	30	0.20	0.67	5–1000	0.9997
					253.0	126.0	25	10				
98	Thiamethoxam	C_8_H_10_ClN_5_O_3_S	[M + H]^+^	1.68	292.0	132.0	22	22	0.46	1.53	5–1000	0.9999
					292.0	211.2	22	12				
99	Thifluzamide	C_13_H_6_Br_2_F_6_N_2_O_2_S	[M + H]^+^	6.53	526.8	148.0	20	25	1.69	5.63	10–1000	0.9993
					526.8	168.0	20	25				
100	Thiophanate-methyl	C_12_H_14_N_4_O_4_S_2_	[M + H]^+^	3.20	343.1	93.0	20	46	0.72	2.39	5–1000	0.9996
					343.1	151.0	20	22				
101	Tolfenpyrad	C_21_H_22_ClN_3_O_2_	[M + H]^+^	6.11	384.2	171.0	30	20	1.13	3.78	5–1000	0.9997
					384.2	197.0	30	18				
102	Triadimefon	C_14_H_16_ClN_3_O_2_	[M + H]^+^	5.74	294.1	69.3	30	20	0.20	0.68	5–1000	0.9988
					294.1	197.2	30	15				
103	Triazophos	C_12_H_16_N_3_O_3_PS	[M + H]^+^	6.19	314.1	118.9	30	35	0.49	1.63	5–1000	0.9996
					314.1	161.9	30	18				
104	Tribenuron-methyl	C_15_H_17_N_5_O_6_S	[M + H]^+^	4.09	396.1	154.9	18	14	0.30	1.00	5–1000	0.9885
					396.1	180.9	18	22				
105	Tridemorph	C_19_H_39_NO	[M + H]^+^	4.29	298.1	57.0	40	28	1.24	4.14	5–1000	0.9997
					298.1	98.0	40	34				
106	Trifloxystrobin	C_20_H_19_F_3_N_2_O_4_	[M + H]^+^	5.94	409.0	145.0	25	25	0.48	1.58	5–1000	0.9994
					409.0	186.0	25	8				
107	Triflumizole	C_15_H_15_ClF_3_N_3_O	[M + H]^+^	4.92	346.0	277.9	13	10	1.75	5.82	10–1000	0.9998
					359.0	139.1	20	35				

**Table 2:** Recovery (RE) values, Precision, Stability and Matrix effect (ME) of 107 Pesticides.

No.	Compound	10 µg/kg		50 µg/kg		100 µg/kg		Intra-day Precision (RSD%, n = 5)		Inter-day Precision (RSD%, n = 5)		Stability (RSD, %)	ME (%)
		Rec. (%)	RSD (%)	Rec. (%)	RSD (%)	Rec. (%)	RSD (%)	10 µg/kg	100 µg/kg	10 µg/kg	100 µg/kg	100 µg/kg	10 µg/kg
1	Abamectin	83.3	8.1	86.0	13.1	87.0	8.2	3.2	2.7	5.4	1.9	6.2	90.0
2	Acephate	71.7	1.0	80.0	2.0	67.0	0.9	0.7	3.1	3.6	6.3	5.4	83.8
3	Acetamiprid	85.0	8.8	96.0	9.0	93.5	6.8	1.6	1.6	2.1	2.8	3.9	97.5
4	Acetochlor	86.7	7.9	104.3	5.9	102.3	3.3	2.1	5.4	6.3	4.6	4.8	99.6
5	Aldicarb	96.7	5.6	92.3	3.7	86.8	0.6	6.2	1.0	4.8	11.7	1.9	92.4
6	Aldicarb-Sulfone	90.0	13.5	93.3	10.4	94.7	6.6	0.6	2.2	7.2	2.7	7.2	91.6
7	Aldicarb-Sulfoxide	85.0	5.5	78.0	4.1	102.0	4.7	0.8	3.6	1.9	10.8	10.8	87.9
8	Ametryn	90.0	2.7	92.3	5.7	102.0	3.6	2.3	7.8	4.5	11.6	3.5	101.4
9	Atrazine	103.3	9.1	92.0	4.1	96.5	5.3	5.7	1.2	2.6	3.8	7.2	102.6
10	Azoxystrobin	63.3	9.1	77.3	2.6	104.7	2.7	1.0	4.3	5.6	4.6	5.6	96.8
11	Bendiocarb	103.3	2.8	104.6	4.3	92.8	0.6	9.3	3.2	10.2	7.2	6.3	104.7
12	Blasticidin-S	96.5	3.1	101.2	1.6	99.8	0.7	5.6	7.6	2.8	6.3	8.2	103.2
13	Boscalid	80.0	10.8	79.0	7.4	77.0	3.3	7.2	4.5	4.6	2.1	6.3	79.8
14	Bupirimate	116.0	8.9	93.7	5.2	93.2	4.3	1.4	6.2	7.9	1.6	4.9	107.2
15	Buprofezin	103.3	5.6	98.0	2.0	85.8	0.9	5.3	1.9	5.3	1.9	5.1	92.4
16	Butralin	91.7	8.3	92.6	0.6	85.0	3.8	5.9	8.2	4.1	2.0	7.2	88.3
17	Carbaryl	103.3	12.9	79.0	5.0	92.0	2.4	2.7	4.1	2.2	3.6	4.1	94.5
18	Carbendazim	92.4	6.3	96.3	3.2	102.6	1.8	0.5	2.0	1.0	5.4	2.8	95.2
19	Carbofuran	113.3	2.5	118.6	6.2	118.3	3.3	9.4	3.3	3.2	8.2	2.2	121.6
20	Carbosulfan	106.3	2.1	98.2	1.9	104.2	4.1	1.6	6.1	5.1	7.5	1.6	98.2
21	Cartap	108.3	2.6	85.6	3.5	89.0	6.1	4.1	2.9	11.3	1.9	4.3	95.1
22	Chlorantraniliprole	100.0	13.2	82.5	4.5	79.0	6.7	6.8	2.5	0.9	3.6	5.3	89.7
23	Chlorfenvinphos	95.0	3.6	88.7	5.5	89.7	3.9	2.4	1.8	2.8	9.2	5.9	92.3
24	Chlorpropham	100.0	5.0	106.7	10.1	75.3	13.6	2.2	5.5	5.6	1.6	11.2	80.6
25	Clodinafop-propargyl	83.3	3.4	86.0	11.0	85.8	7.3	1.0	4.9	8.4	5.5	9.7	87.1
26	Clothianidin	88.3	8.6	90.3	3.5	104.5	6.3	1.3	5.6	7.6	8.0	3.8	93.5
27	Cyantraniliprole	75.0	13.3	77.5	4.8	83.8	5.6	3.0	8.2	3.5	7.9	4.9	85.9
28	Cyazofamid	100.0	3.1	86.7	2.4	94.5	1.9	1.8	7.1	6.1	3.6	7.4	96.1
29	Cymoxanil	115.0	11.5	99.0	6.0	96.3	3.5	2.6	2.3	4.8	2.1	6.6	104.2
30	Cyproconazole	96.7	2.9	87.7	1.7	103.2	6.1	2.8	1.1	6.6	9.7	5.9	95.3
31	Cyprodinil	80.0	12.7	78.3	10.3	88.9	11.8	4.2	1.0	7.4	4.8	8.7	88.9
32	Cyromazine	90.7	6.8	95.4	3.2	94.6	5.7	3.1	2.0	2.5	5.1	9.2	90.2
33	Diazinon	96.7	2.9	97.3	0.5	96.7	1.6	6.5	2.4	4.6	3.2	7.4	100.4
34	Diethofencarb	100.0	5.0	97.3	4.2	114.0	7.0	5.1	3.5	1.9	1.9	5.4	106.2
35	Difenoconazole	75.0	6.7	75.7	2.5	87.8	4.4	3.1	6.1	8.4	5.6	3.2	90.3
36	Dimethoate	95.0	2.6	97.0	2.0	92.2	0.8	5.0	2.2	7.6	4.9	2.6	94.8
37	Dimethomorph	86.7	3.3	92.0	1.0	90.3	4.9	6.2	0.8	3.8	8.5	2.1	91.7
38	Diniconazole	76.7	3.7	79.0	4.8	76.3	7.3	1.8	1.2	5.4	4.0	4.4	86.3
39	Dinotefuran	88.3	8.6	92.0	3.9	103.0	6.5	0.9	1.9	2.9	3.9	7.0	88.6
40	Diuron	75.0	6.6	76.0	4.8	78.3	4.4	2.2	3.7	0.8	2.1	6.2	87.2
41	Epoxiconazole	72.0	4.0	82.5	9.4	81.5	7.1	2.3	2.6	11.6	6.3	5.9	89.1
42	Ethion	78.3	13.2	96.3	4.3	89.2	7.3	1.6	5.4	12.3	9.7	7.6	93.2
43	Fenbuconazole	80.0	2.2	83.5	5.6	81.2	10.3	1.8	5.7	2.2	8.2	3.9	91.7
44	Fenhexamid	111.0	11.2	87.3	11.0	75.2	7.1	3.3	6.1	2.0	5.1	9.8	92.8
45	Fenobucarb	105.0	8.2	90.7	9.1	91.7	4.7	2.9	3.3	6.5	4.2	11.7	99.7
46	Flonicamid	101.7	2.8	93.0	2.8	103.0	2.7	4.2	1.8	1.9	1.2	7.9	103.2
47	Fluazifop-butyl	88.3	6.5	95.7	2.1	84.5	4.3	3.6	1.1	7.1	2.9	5.9	96.5
48	Fluazinam	81.7	9.3	82.0	12.2	76.7	5.5	5.1	2.6	6.3	3.5	4.2	86.7
49	Fluopicolide	83.3	3.4	93.3	7.5	100.8	3.6	1.8	3.7	1.8	7.4	1.8	96.3
50	Flusilazole	93.3	3.0	93.0	2.1	91.8	5.8	1.7	8.6	7.7	6.1	4.1	97.2
51	Flutriafol	88.3	3.2	91.0	4.7	106.8	5.1	2.2	9.0	6.5	9.8	3.1	95.4
52	Fosthiazate	96.7	7.9	96.3	0.6	93.8	2.1	1.3	5.7	13.6	4.2	5.8	100.7
53	Haloxyfop-methyl	91.7	3.1	88.3	3.6	87.7	5.7	3.3	3.1	5.8	6.6	4.2	90.6
54	Hexaconazole	86.7	12.0	92.7	8.3	101.7	6.4	2.9	6.8	6.2	1.5	1.9	95.7
55	Hexythiazox	75.0	6.6	89.0	2.9	91.0	10.0	8.2	7.2	4.9	4.6	7.4	77.3
56	Imazalil	68.3	11.1	80.0	8.0	70.2	3.1	7.6	1.9	7.5	5.1	4.8	85.9
57	Imidacloprid	76.7	3.7	86.3	2.6	102.3	2.2	1.4	4.4	4.6	8.4	4.6	94.3
58	Indoxacarb	88.3	3.2	93.7	4.0	82.3	9.9	6.2	4.7	1.8	7.4	5.0	91.6
59	Iprodione	90.0	5.5	72.0	2.7	86.8	9.2	9.4	2.8	4.2	3.3	2.7	87.8
60	Isocarbophos	80.0	12.5	96.0	8.1	96.3	6.2	8.0	3.9	1.3	2.9	6.0	98.4
61	Isoprocarb	106.7	2.7	95.0	4.8	93.7	1.3	7.2	6.5	2.6	6.1	4.8	103.2
62	Isoprothiolane	115.0	11.5	105.0	4.3	89.2	7.7	4.4	2.2	5.8	3.2	7.1	101.6
63	Isoproturon	101.7	2.8	94.7	0.6	106.0	3.1	4.6	1.3	7.1	1.9	4.2	99.1
64	Malathion	80.0	2.3	87.3	1.1	89.5	2.1	2.8	7.2	6.3	7.8	6.6	96.7
65	Metalaxyl	90.0	5.5	97.3	2.1	93.5	1.4	3.9	4.5	6.9	6.4	5.9	92.5
66	Metconazole	76.7	13.5	76.0	5.9	73.2	5.5	4.1	6.1	5.5	1.4	8.2	76.3
67	Methamidophos	76.7	3.7	81.0	2.4	96.7	3.0	3.2	2.8	1.8	0.8	8.0	88.2
68	Methomyl	88.3	11.7	95.0	4.5	115.8	3.2	6.1	7.0	8.2	1.9	10.6	98.4
69	Methoxyfenozide	85.0	11.7	87.7	9.2	90.3	3.3	5.8	8.2	3.6	2.8	2.1	91.5
70	Metolachlor	100.0	1.8	97.7	3.1	95.8	2.6	4.6	9.1	5.8	4.6	1.9	96.7
71	Metribuzin	91.7	6.3	94.0	6.5	93.5	3.2	0.8	2.4	6.2	7.1	1.5	89.7
72	Myclobutanil	118.0	6.4	93.7	2.2	105.7	4.9	1.1	3.3	2.9	7.3	4.1	102.7
73	Omethoate	81.7	3.5	76.3	5.4	94.2	3.2	2.3	5.1	7.1	5.9	3.3	88.2
74	Paclobutrazol	95.0	5.2	90.0	6.7	98.8	4.3	2.9	2.9	4.4	4.5	2.3	95.4
75	Penconazole	100.0	5.0	95.0	2.8	99.5	3.5	2.2	4.6	1.8	2.9	6.5	98.7
76	Phenthoate	85.0	5.8	99.7	1.2	93.0	1.9	5.1	4.1	2.6	8.7	4.9	94.2
77	Phorate	86.7	13.8	105.3	5.5	110.1	5.1	1.6	2.9	3.7	12.6	7.3	102.1
78	Phorate-sulfone	80.0	6.2	100.7	4.6	103.0	3.2	1.1	4.1	5.6	5.9	5.4	97.4
79	Phorate-sulfoxide	90.0	5.5	95.7	1.2	98.8	1.0	7.5	6.2	4.4	4.8	6.1	92.7
80	Phoxim	88.3	6.5	98.7	1.5	100.0	1.1	8.6	5.7	4.4	8.9	9.7	95.1
81	Piperonyl butoxide	81.7	3.5	85.0	3.1	90.7	4.2	8.3	6.0	2.8	4.6	8.2	87.3
82	Pirimiphos-methyl	85.0	4.1	95.7	3.7	106.1	3.0	1.9	2.8	7.6	7.6	8.9	89.6
83	Prochloraz	89.6	1.4	91.6	0.9	96.3	2.5	6.7	4.9	3.6	1.6	4.9	92.3
84	Profenofos	91.7	3.1	90.0	5.7	85.5	5.1	3.2	6.6	5.9	5.2	4.7	92.5
85	Prometryn	90.0	3.8	94.3	0.6	109.5	1.6	4.1	7.4	5.6	1.3	5.4	94.8
86	Propham	116.6	2.4	89.3	6.5	92.2	3.6	8.8	2.9	3.8	5.6	8.2	108.2
87	Propiconazole	83.3	6.9	87.0	3.0	98.7	6.9	8.4	3.6	4.2	4.3	1.7	95.1
88	Propoxur	101.7	10.2	90.0	8.9	103.5	1.0	6.2	1.9	1.9	6.3	4.6	102.7
89	Pyridaben	80.0	1.9	95.3	2.4	103.8	11.2	8.5	1.3	4.4	2.6	6.2	94.3
90	Semiamitraz	85.6	8.9	89.3	2.6	87.6	3.5	1.9	3.5	7.5	1.9	3.9	89.7
91	Sethoxydim	123.0	8.4	101.7	4.0	90.3	4.0	2.7	4.9	6.3	4.6	7.4	107.3
92	Simazine	91.7	3.1	89.0	6.0	89.8	10.2	3.6	8.2	6.9	2.5	1.5	90.6
93	Spirodiclofen	85.0	12.9	97.3	2.4	82.5	7.3	1.8	7.1	5.5	1.0	8.4	87.5
94	Tebuconazol	95.0	5.2	80.0	7.6	81.0	3.2	2.2	7.5	8.0	6.8	5.6	89.4
95	Tebufenozide	111.7	8.5	99.3	7.4	84.7	2.3	2.2	2.9	10.6	7.2	9.2	98.6
96	Tetraconazole	85.0	5.8	91.7	1.6	100.8	4.2	6.3	6.2	2.8	2.2	9.1	92.5
97	Thiacloprid	88.3	3.2	93.7	0.6	92.7	2.1	4.1	5.9	4.5	2.6	4.8	93.6
98	Thiamethoxam	88.3	3.2	90.0	2.9	92.7	1.1	3.9	3.8	11.3	5.1	4.3	97.4
99	Thifluzamide	71.7	4.0	103.7	8.2	89.5	8.7	5.7	1.6	1.7	4.3	2.2	88.7
100	Thiophanate-methyl	88.3	3.2	92.0	3.7	92.2	12.4	6.2	4.6	12.8	6.2	2.6	91.8
101	Tolfenpyrad	83.3	9.1	80.0	3.3	84.2	2.0	2.4	1.5	3.3	1.8	9.0	86.9
102	Triadimefon	91.7	3.1	91.7	4.9	92.0	3.8	3.3	2.3	5.7	4.1	8.1	96.5
103	Triazophos	100.0	5.0	90.7	9.2	84.7	12.7	0.8	6.2	4.9	2.9	6.1	103.6
104	Tribenuron-methyl	100.0	3.3	73.3	13.2	82.8	2.1	1.9	5.5	8.1	3.6	7.2	89.7
105	Tridemorph	105.0	4.7	81.7	11.4	79.5	7.7	5.1	1.9	6.2	1.1	3.6	91.8
106	Trifloxystrobin	91.7	3.1	95.3	0.6	83.3	2.2	3.3	2.1	5.0	3.2	4.9	90.2
107	Triflumizole	90.0	5.5	86.3	8.8	95.2	4.9	2.9	4.6	4.1	5.1	6.2	93.6
